# Etymologia: **schistosomiasis**

**DOI:** 10.3201/eid1310.071310

**Published:** 2007-10

**[shis”-, skis” to-so-mi’ə-sis], from the Greek—s*khistos* (split) and *soma* (body)**

Infection of the blood with a parasite of the genus *Schistosoma*. Originally thought a single organism with a split body, the parasite was eventually recognized as having male and female forms. Three main species cause human infection: *S. haematobium, S. mansoni,* and *S. japonicum*. Each species has its own range of host snails. The parasite releases eggs containing larvae through feces or urine; if the eggs reach water, the larvae are released and may penetrate a snail. A very large number of larvae are then produced inside the snail and released back into the water. Infection is acquired through skin contact with contaminated water.

Schistosomiasis, which leads to chronic hepatic and intestinal fibrosis of the urinary tract, was first identified in Egypt in 1851 by German pathologist Theodor Bilharz and is also called bilharzia. Approximately 160 million persons throughout the world are infected, particularly in Africa, the Middle East, South America, and Southeast Asia.

